# Neuroinflammation in Parkinson’s disease and its potential as therapeutic target

**DOI:** 10.1186/s40035-015-0042-0

**Published:** 2015-10-12

**Authors:** Qinqin Wang, Yingjun Liu, Jiawei Zhou

**Affiliations:** Institute of Neuroscience, State Key Laboratory of Neuroscience, CAS Center for Excellence in Brain Science, Shanghai Institutes for Biological Sciences, Chinese Academy of Sciences, 320 Yueyang Road, Shanghai, 200031 China

**Keywords:** Parkinson’s disease, Neurodegeneration, Glial cells, Neuroinflammation

## Abstract

Parkinson’s disease (PD), the second most common age-associated neurodegenerative disorder, is characterized by the loss of dopaminergic (DA) neurons and the presence of α-synuclein-containing aggregates in the substantia nigra pars compacta (SNpc). Chronic neuroinflammation is one of the hallmarks of PD pathophysiology. Post-mortem analyses of human PD patients and experimental animal studies indicate that activation of glial cells and increases in pro-inflammatory factor levels are common features of the PD brain. Chronic release of pro-inflammatory cytokines by activated astrocytes and microglia leads to the exacerbation of DA neuron degeneration in the SNpc. Besides, peripheral immune system is also implicated in the pathogenesis of PD. Infiltration and accumulation of immune cells from the periphery are detected in and around the affected brain regions of PD patients. Moreover, inflammatory processes have been suggested as promising interventional targets for PD and even other neurodegenerative diseases. A better understanding of the role of inflammation in PD will provide new insights into the pathological processes and help to establish effective therapeutic strategies. In this review, we will summarize recent progresses in the neuroimmune aspects of PD and highlight the potential therapeutic interventions targeting neuroinflammation.

## Introduction

Parkinson’s disease (PD) is an age-related neurodegenerative disorder characterized clinically by resting tremor, slowness of movement, rigidity and postural instability and pathologically by the progressive loss of dopaminergic (DA) neurons in the substantia nigra pars compacta (SNpc) [1–4]. Deposition of protein aggregates containing α-synuclein (termed Lewy bodies) is evident in multiple brain regions of advanced PD patients [5]. The etiology of PD has not yet been fully understood. Although a variety of possible pathogenetic mechanisms have been proposed over the years, including excessive release of oxygen free radicals during enzymatic dopamine breakdown, impairment of mitochondrial function, loss of trophic support, abnormal kinase activity, disruption of calcium homeostasis, dysfunction of protein degradation and neuroinflammation, the pathogenesis of PD is still largely uncertain [6–8]. However, emerging evidence indicates that sustained inflammatory responses, T cell infiltration and glial cell activation are common features of both human PD patients and animal models of PD and play vital roles in the degeneration of DA neurons [9, 10], which suggests the possibility of developing potential therapies for PD by targeting the inflammatory processes. In this review, we will focus on the role of inflammation in the progression of PD and the potential application of anti-inflammatory medications in the treatment of this devastating disorder.

### Microglia-mediated inflammation in PD

Microglia is one of the major cell types which are involved in the inflammatory responses in the central nervous system (CNS) [11]. In 1988, McGeer et al. showed the presence of reactive microglia in the SNpc of human post-mortem brain tissue, which is the evidence revealed for the first time suggesting the involvement of neuroinflammation in PD pathogenesis [12]. Positron emission tomography (PET) studies also indicate that there is pronounced activation of microglia in various regions of PD brain [13, 14]. Moreover, activation of microglia in the SNpc and striatum is profound in various types of PD animal models [15–17]. Further biochemical analysis reveals higher levels of pro-inflammatory mediators including tumor necrosis factor-α (TNF-α), interleukin-1β (IL-1β) and interferon-gamma (IFN-γ) in the midbrain of PD patients. These data strongly suggest the involvement of immune components in PD pathogenesis.

Under physiological conditions, the quiescent state of microglia is maintained by a variety of immunomodulators, such as CX3CL1, CD200, CD22, CD47, CD95 and neural cell adhesion molecule (NCAM), which are produced mainly by neuronal cells [18–26]. Interestingly, the receptors for these molecules are almost exclusively expressed by microglia in the CNS, indicating the critical role of neuron-microglia interactions in the regulation of neuroinflamamtion [18–26]. For instance, CX3CL1-CX3CR1 signaling negatively regulates microglial activation and protects DA neurons from degeneration induced by neurotoxins [27, 28]. Deficiency of CX3CL1 or CX3CR1 in vivo results in increased neurotoxicity induced by systemic lipopolysaccharide (LPS) treatment and enhanced cell death of DA neurons in the SNpc of animal PD models [28, 29]. Likewise, dysfunction of CD200-CD200R signaling also increases the activation of microglia and exacerbates the degeneration of DA neurons in rat PD models [30, 31].

It has been proposed that activated microglia may be beneficial to the host, at least in the early phase of neurodegeneration process [12, 32–34]. For instance, it has been shown that suppression of Jmjd3, which is essential for M2 microglia polarization, in the substantia nigra (SN) *in vivo* dramatically causes microglial overactivation and exacerbated dopamine (DA) neuron death in a PD animal model [35], indicating a protective role of M2 microglia in this process. However, long-term over-activation of microglia in the PD brain significantly up-regulates the expression of a large group of pro-inflammatory cytokines including TNF-α, IL-1β, interleukin-6 (IL-6) and IFN-γ, which contribute to the acceleration of nigral DA neuron degeneration [36, 37]. As the disease progresses, molecules such as α-synuclein, ATP and metalloproteinase-3 (MMP-3) released from the degenerating DA neurons will further enhance microglia activation, amplify the neuroinflammatory responses in the brain, and result in the deterioration of the neurodegenerative processes [11, 38] forming a vicious cycle of neurodegeneration. Activated microglia accumulate around the α-synuclein-positive aggregates in many regions of PD brain [39]. These cells are likely activated by over-produced [38, 40], mutants or misfolded α-synuclein leading to increases in the production and release of the pro-inflammatory cytokines [38, 41, 42]. Thus, the neurotoxicity induced by excessive or misfolded α-synuclein may be partially caused by microglia-mediated inflammatory responses.

ATP, a purinergic neurotransmitter, is also able to robustly modulate various functions of microglia [43, 44]. The migration of microglia to the injured and inflammatory areas is controlled by ATP released from the damaged neurons and neighboring astrocytes [44]. In addition, ATP binds to the P2Y receptor which is mainly expressed by microglia in the brain and induces the production of high levels of IL-1β, TNF-α and nitric oxide (NO) [45]. Another protein produced by degenerating neurons is MMP-3 which also plays important roles in the regulation of the activation states of microglia, at least *in vitro*. Overexpression of MMP-3 in microglia-neuron co-cultures induces prominent activation of microglia and increased the oxidative stress reaction. In contrast, MMP-3^−/−^ mice administrated with N-methyl-4-phenyl-1,2,3,6-tetrahydropyridine (MPTP) display attenuated nigrostriatal DA neuronal degeneration, microglial activation, and superoxide generation [46]. These data support the notion that microglia is a major player in neuroinflammation in the context of PD pathogenesis and MMP-3 plays a pivotal role in dopaminergic neuronal degeneration.

### Microglia activation phenotypes in PD

Mounting evidence indicates that microglia has two alternative activation phenotypes, termed the M1 (pro-inflammatory) phenotype and the alternative M2 (anti-inflammatory) phenotype. These different activation statuses of microglia are characterized by secretion of different arrays of cytokines [47]. It has been demonstrated that LPS/IFN-γ treatment induces M1 activation, while IL-4/IL-13 treatment triggers M2 activation in microglia. The classical M1 activation of microglia is featured by the production of pro-inflammatory cytokines, including TNF-α, IL-1β, IL-6, IL-12, and other cytotoxic molecules such as superoxide, NO and reactive oxygen species (ROS), contributing to the amplification of the pro-inflammatory responses during injuries and infections. Conversely, M2 microglia plays an immunosuppressive role by antagonizing the classic M1 microglia and promoting tissue repair. The M2 microglia produces a variety of cytokines with anti-inflammatory property, such as IL-4, IL-13, IL-10, and TGF-β. The different activation forms of microglia can be distinguished by their characteristic gene expression pattern. For example, Arg1, FIZZ1 (also known as RELM-α), Chi3l3 (also known as YM1) and CD206 were expressed in mouse M2 phase microglia [48]. Expression of Arg1, FIZZ1 and Chi3l3 may be regulated by cytokines, since their levels are significantly increased in primary cultured microglia or the striatal and frontal cortical regions of mouse brain following IL-4 stimulation [49].

What factors affect M1/M2 microglia phenotype in the context of PD? Mis-folded proteins and environmental toxins induce the activation of microglia toward M1 phenotype in PD animal models [38, 50, 51]. Chronic MPTP administration leads to progressive reduction of CD206 expression, which suggests the down-regulation of M2 phase activation of microglia in the progression of PD [47]. Conversely, IL-4 treatment up-regulates microglial expression of histone H3K27me3 demethylase (Jmjd3) which is involved in the regulation of epigenetic modification of chromosomes and contributes to various human diseases. Expression levels of M2 marker genes, such as Arg1 and CD206, are significantly down-regulated after Jmjd3 knockdown in N9 microglia cell line, indicating the essential role of Jmjd3 for M2 microglia polarization. Knockdown of Jmjd3 *in vivo* exacerbates the DA neuron loss in the SNpc of MPTP-induced mouse PD model by revoking M2 activation of microglia [35].

**Fig. 1:**
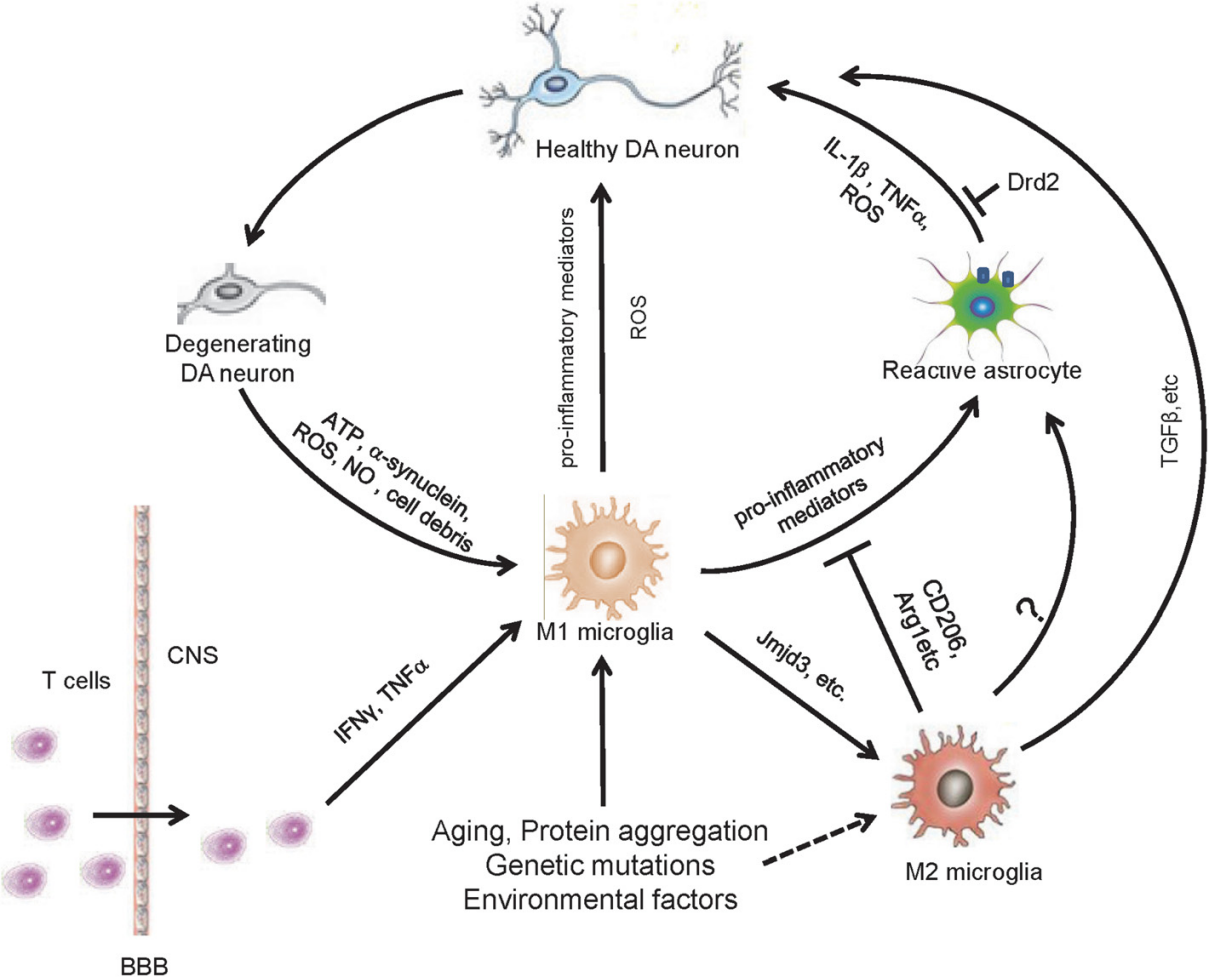
Diagrammatic representation of inflammatory mechanisms involved in PD pathogenesis. Microglia become activated M1 phenotype in PD under pathological conditions such as protein aggregation, gene mutations, environmental factors and cytokines released from infiltrated T cells. The pro-inflammatory mediators from M1 microglia activate astrocytes leading to elevated production of proinflammatory factors, nitric oxide and superoxide radical, contributing to degeneration of DA neurons. The molecules released from degenerative DA neurons can further cause activation of glia and enhanced inflammatory response. At certain stage of PD, subpopulation of microglia may become activated M2 phenotype releasing anti-inflammatory factors, including TGF-β, and exert a neuroprotective effect in PD

Conditioned medium (CM) from M1 phase N9 microglia results in increased death of DA neurons, whereas CM mixture from both M1 and M2 cells reverses the neurotoxicity elicited by the M1-CM [35]. Previous investigations indicated that a majority of the activated microglia express M2 associated genes at the early stages following injury in various models. M1 signature genes, however, gradually become predominant in later stages [32]. These interesting observations suggest that it is important to balance different microglia activation phenotypes in PD (Fig. 1). It appears to be a promising strategy to intervene the progression of PD by manipulating the transition of microglia activation statuses. Even though current data suggest differential role of M1 and M2 in the pathogenesis of PD in its animal models, similar results from patients are lacking. Future work should warrant research in this aspect.

### Astrocyte-mediated neuroinflammation in PD

A great body of studies show that astrocytes also play vital roles in the neuroinflammatory processes in PD. Like microglia, astrocytes respond to the inflammatory stimulations such as LPS, IL-1β and TNF-α by producing pro-inflammatory cytokines both *in vitro* and *in vivo* [11, 52]. Reactive astrogliosis characterized by the increased expression levels of glial fibrillary acidic protein (GFAP) and hypertrophy of cell body and cell extensions have been reported in various PD animal models. Importantly, astrogliosis also exists in the affected brain regions of patients with PD, indicating the possible involvement of astrocytes in the immune processes in PD [53].

It has been observed that astrocytic responses are relatively slower than microglial activation after stimulations. Microglia may initiate the inflammatory responses after immune stimulations such as LPS treatment and α-synuclein aggregation. Astrocytes are then activated by a variety of molecules including pro-inflammatory mediators released from activated microglia and these immunosignals are further amplified by astrocytes [11]. Uncontrolled neuroinflammation caused by the synergic activation of microglia and astroyctes ultimately contributes to the enhanced death of DA neurons in the SNpc during neurodegeneration [11, 54]. The expression levels of TNF-α and IL-6 of primary cultured astrocytes are increased significantly after α-synuclein treatment *in vitro* [55]. Specific overexpression of mutant α-synuclein in astrocytes causes widespread astrogliosis, microglial activation and degeneration of DA neurons and motor neurons in mice [56].

One of the long-standing questions in PD research is how the neuroinflammation is developed and the roles of astrocytes in this process. Human post-mortem and animal experimental results suggest a progressive decline of the dopaminergic neuronal system with age [57]. Whether the reduction of Drd2 in aging brain has significant impact on brain function and ultimately contribute to the development of early/mid-stage PD remains poorly understood. This reduction primarily reflects the changes in neuronal cells. In sharp contrast, the contribution from glial Drd2 in overall levels is minor. Indeed, our own studies have revealed that astrocytic Drd2 is of very low abundance in overall levels of Drd2 in the striatal tissue [58]. Given that astroglial and microglial cells are the ‘sensors’ in the brain which constantly monitor brain activities, it is likely that glial Drd2 is more vulnerable to microenvironment changes, despite that glial Drd2 abundance is very low in the brain. Nevertheless, the biological consequence of glial Drd2 loss may be significant. Ablation of astrocytic Drd2 causes dramatic reduction of anti-inflammation protein alphaB-crystallin in the CNS [58, 59]. Thus, glial Drd2 may be an important player in the maintenance of immune homeostasis (Fig. 1). It is plausible that down-regulation of Drd2, presumably including those in glial cells, in aging brain compromise the immune homeostasis contributing to PD pathogenesis. Future studies may be required to develop new technology that can specifically label and visualize glial dopamine receptors in aging and PD brain.

The idea that Drd2 plays important roles in the modulation of neuroinflammation is supported by a recent study in which Drd2 activation by Drd2 agonists quinpirole and ropinirole reduces expression levels of IL-1β and monocyte chemoattractant protein-1 as well as microglia/macrophages activation in experimental intracerebral hemorrhage brain injury model [60]. These results further suggest the anti-inflammatory effects of Drd2 in certain CNS disorders.

### PD-associated genes and neuroinflammation

Increasing evidence indicates that some PD-associated genes are involved in the regulation of immune responses of microglia and astrocytes in the CNS. One of such genes is α-synuclein (SCNA), the missense mutations of which results in a familial form of PD [61]. Abnormal accumulation of α-synuclein in neuronal cytoplasm and neurites is one of the pathological hallmarks of PD [62]. Wild-type or pathogenic forms of α-synuclein induces pronounced microglial activation *in vitro* [63]. Furthermore, oligomers of α-synuclein could elicit microglia responses through the activation of toll-like receptor 2 (TLR2)-mediated signaling [64]. In α-synuclein transgenic mice, there is prominent activation of microglia and upregulation of TLRs in the brainstem and SNpc [65, 66].

In the last several years, the common variations in leucine-rich repeat kinase 2 (LRRK2) gene have been identified as risk factors of both familial and sporadic PD [67–70]. Inflammatory stimuli such as LPS could drastically enhance the expression levels of LRRK2 in primary cultured microglia [71], while knockdown of LRRK2 reduces LPS-induced TNF-α and iNOS production and also decreases the activation of nuclear factor κ B (NF-κB) transcriptional activity in microglia [72]. Moreover, the expression levels of pro-inflammatory cytokines are higher in microglia isolated from LPS-challenged transgenic mice overexpressing R1441G mutation of LRRK2 than in microglia from LPS-treated WT mice [73]. These data suggest that LRRK2 regulates microglia activation thus might contribute to the progression of PD through neuroinflammatory pathways.

Of note, Lrrk2 has been shown to play crucial roles in periphery inflammation. LRRK2 is relatively abundant in peripheral blood mononuclear cells and macrophages in the immune system. Expression levels of LRRK2 are significantly increased in the process of THP-1 monocyte-to-macrophage differentiation following IFN-γ treatment [74, 75]. LRRK2 deficiency results in enhanced susceptibility to experimental colitis in mice [76], indicating the important role of LRRK2 in periphery inflammation and its potential association with PD.

Parkin is another PD-associated gene which encodes an E3-ubiquitin ligase. Mutations in *parkin* are the most common causes of recessively inherited PD [77]. Systemic LPS treatment induces more prominent degeneration of DA neurons in the SNpc of *parkin* knockout mice, comparing with littermate controls [78]. Aged *parkin* knockout mice display increased astrogliosis in the striatum and aberrant microglial activation in the midbrain [79]. Co-culture of microglia from *parkin* knockout mice and wild-type neurons increase sneuronal vulnerability to rotenone toxicity [80], suggesting that *parkin*-null microglia produce and release soluble factors that are detrimental to neuronal cells. Indeed, these microglia express higher levels of pro-inflammatory cytokines such as TNF-α, IL-6 and iNOS after LPS challenge [81], implicating that parkin plays an important role in the regulation of PD-associated inflammation.

Missense mutations in the PTEN-induced putative kinase1 (PINK1) gene cause the early-onset, recessively inherited form of PD [82]. Under pathological conditions, PINK1 directly phosphorylates parkin to enhance its activity and causes mitochondria damages [83]. PINK1 is also involved in the regulation of inflammatory cytokine production. Under basal condition and following systemic LPS treatment, mice null for *PINK1* produce higher levels of pro-inflammatory cytokines including IL-1β, IL-12 and TNF-α [84, 85]. The underlying molecular mechanisms of PINK1’s effect remain elusive. PINK1 may regulate IL-1β-mediated NF-κB activity through interacting with IL-1 receptor-associated kinase 1 (IRAK1) and toll-interacting protein (Tollip) [86].

It has been shown that DJ-1, one of PD genes, is mainly expressed in astrocytes and microglia in human brain and the expression levels of DJ-1 are strongly upregulated in reactive astrocytes in PD patients [87]. Astrocytes from *DJ-1* knockout mice produce higher levels of cyclooxygenase-2 (COX2) and IL-6 following LPS treatment [4]. Primary neurons plated on *DJ-1*-null astrocytes shows severe apoptosis following LPS treatment, which indicates that loss of DJ-1 might contribute to PD pathogenesis by astrocyte-mediated neuroinflammatory response [4]. Likewise, microglia with DJ-1 knockdown also exhibits hyper-responsiveness to LPS [88]. Taken together, it is highly likely that mutation, down-regulation or overexpression of PD associated genes perturb the expression of pro-inflammatory cytokines in glial cells after LPS treatment. The question that how PD genes regulate inflammatory responses in PD should be addressed in the future.

### Peripheral immune cell-mediated inflammation in PD

Under physiological conditions, the peripheral immune cells such as T- and B-lymphocytes are hardly detectable in the CNS. Following infections or tissue injuries, blood monocytes and tissue-resident macrophages are quickly activated and secret an array of inflammatory cytokines like IL-1β, TNF-α and IL-6 as well as chemokines. These cytokines and chemokines could enter the brain and stimulate microglia to initiate the neuroinflammatory reactions [89]. The CNS has been considered as immunologically privileged because of the existence of blood brain barrier (BBB). It is known that the neurovascular unit is formed by tight junctions between endothelial cells and the surrounding components of the CNS, including pericytes, astrocytes and the basement membrane. BBB limits the entrance of pathogens and peripheral immune cells into the brain parenchyma. BBB breakdown leads to increased infiltration of peripheral immune cells into the CNS, which has been identified as one of the major contributing factors for neurodegenerative diseases including PD [90, 91]. There are dramatically morphological changes of endothelial cells in the SNpc of PD brain, suggesting the possible dysfunction of BBB in the pathological processes of PD [91–93]. Several studies show a strong correlation between the BBB disruption and loss of DA neurons in mice by intranigral injection of vascular endothelial growth factor (VEGF) which is a potent inducer of BBB damages [94, 95]. Furthermore, the expression levels of VEGF are drastically increased in both PD patients and the MPTP-induced mouse PD model [94].

The number of activated microglia and infiltrated B and T-lymphocytes is significantly increased in the SNpc of AAV-mediated α-synuclein overexpressing mice. Moreover, nitrated α-synuclein can get through the BBB, enter cervical lymph node causing activation of T-lymphocytes and increases in the expression of the class II major histocompatibility complex in MPTP-treated mice. Transplantation of T cells from mice immunized with nitrated α-synuclein into the brain significantly exacerbates the neuroinflammatory responses and degeneration of DA neurons. Conversely, mice deficient in T- and B-lymphocytes are more resistant to the neurotoxicity caused by MPTP intoxication [16, 96]. Furthermore, MPTP-induced neurotoxicity is markedly attenuated in the SNpc of *CD4*^*−/−*^ mice. Peripheral inflammation caused by ulcerative colitis increase LPS-induced DA neuron degeneration, microglial activation, production of pro-inflammatory cytokines and permeability of the BBB [97]. These data indicate that there is a close relationship between the peripheral immune system and the progression of PD. A better understanding of the relationship between the CNS and immune system and the molecular mechanisms underlying their crosstalk may help to further clarify the pathological processes of PD.

It has been reported that systemic infection could contribute to the etiology and the progress of PD [98, 99]. Indeed, the main causes of death in PD patients are respiratory infections [98]. Gastrointestinal infections could lead to a worsening of PD [100, 101]. Moreover, infection of *helicobacter pylori* (HP), a bacterium found on the luminal surface of the gastric epithelium, is associated with clinical diagnosis and worse severity of motor function in PD patients [102, 103]. These data indicate a link between systemic infection and PD. It would be very interesting to see whether control of systemic infections helps to reduce the exacerbation of PD symptoms and delay the disease progression.

### Anti-inflammatory therapies in PD

Given the important roles of neuroinflammation in the initiation and progression of PD, it is desirable to develop intervening therapies for this devastating disorder by targeting the inflammatory pathways mediated by activated glial cells. For example, soluble TNF-α is known to contribute to the progressive degeneration of DA neurons in rodents induced by stereotactic injection of 6-hydroxydopamine (6-OHDA) or LPS. Overexpression of dominant-negative TNF-α specifically inhibits TNF signaling in the SNpc and attenuates activation of microglia, thereby reducing loss of DA neurons and improving locomotor ability in 6-OHDA-induced rat PD model [104, 105].

Minocycline is a semi-synthetic, second-generation tetracycline analog, which, as a lipophilic molecule, can easily get through the BBB and has been reported to have anti-inflammatory and neuroprotective properties in multiple inflammation-related neurological diseases [106, 107]. Minocycline treatment effectively protects DA neurons from degeneration and decreases glial cell activation in the SNpc of LPS and 6-OHDA challenged mice [108, 109]. Administration of minocycline also blocks MPTP-induced neurotoxicity of DA neurons *in vivo* by directly or indirectly inhibiting the phosphorylation of p38 MAPK, which is a key regulator of the expression of inflammatory genes [110]. It is often disappointing to note that, although there are many encouraging studies, the therapeutic potential of minocycline in PD is still controversial. In addition to beneficial effect of mincycline, it is also found to exacerbate the loss of DA neurons and behavior deficits in MPTP-induced rodent and non-human primate models [111]. The reason for these discrepancies is not clear. One of the probabilities is that it may due to the different drug dosages and durations used in different studies.

Besides minocycline, some other compounds with anti-inflammatory properties also show significant protective functions for DA neuron in PD models. Glucocorticoids are well known for their broad ranges of anti-inflammatory effects and have been widely used in clinical studies for brain inflammation. Glucocorticoid receptor expressed on microglia is found to regulate the expression of the transcription factors such as NF-κB and activator protein-1 (AP-1) which are key regulators of inflammatory gene expression [112, 113]. Administration of the synthetic steroid dexamethasone shows a protective effect for DA neurons and decreased the activation of glial cells in the SNpc of MPTP- or LPS-treated mice [112, 114]. Moreover, opioid antagonist naloxone has been shown to inhibit LPS-induced activation of microglia and expression of pro-inflammatory cytokines in vitro, attenuating neuronal loss. The compound dramatically reverses the acute dystonia and parkinsonism following general anaesthesia [115, 116].

Several non-steroidal anti-inflammatory drugs (NSAIDS), such as aspirin, salicylic acid (SA), ibuprofen and celecoxib, have also been shown to have protective effects on DA neurons in PD [117–120]. Aspirin is a potent inhibitor of COX2, which plays important roles in the processes of neuroinflammation and neuronal degeneration [121]. Aspirin promotes the resolution of inflammation [122] and prevents dopamine depletion in the striatum in MPTP-induced mouse and 6-OHDA-induced rat PD models [117]. SA is a metabolite product of aspirin, which is widely used for the treatment of myocardial infarction, cardiovascular diseases [118]. SA administration in MPTP mice alleviates the neurotoxin-induced behavioral impairments as well as dopamine depletion [118, 123]. Similarly, ibuprofen and celecoxib also show protective roles for DA neurons in MPTP- and 6-OHDA-induced PD models [119, 120]. Long-term use of NSAIDs, especially the use of ibuprofen, decreased PD risk by 21 %, suggesting high therapeutic potentials of NSAIDs for PD [120, 124, 125].

### Concluding remarks

Animal experiments and clinical studies have generated an array of evidence supporting the involvement of inflammation in the progression of PD (Fig. 1). However, the precise role of inflammation in PD is still not fully understood. Moreover, the inflammatory responses in PD refer to both glial activation and peripheral immune cell infiltration, but the relationship between these two different inflammatory pathways is still unclear. These issues significantly hamper the development of PD-modifying therapeutics by targeting inflammatory pathways. Moreover, the different forms of microglia activation further increase the difficulty and complexity of manipulation of microglial responses in PD. A better understanding of how these two activated phenotypes of microglia contribute to PD progression may provide a basis for future drug discoveries. So far, many candidate drugs targeting inflammation in PD have been developed and tested. Although some of them have been reported to attenuate the behavior deficits and loss of DA neuron in PD animal models, the clinical studies of these candidates only show moderate effects. Future studies should focus on identifying more specific drug targets by extending the understanding of fundamental processes of inflammation at different stages of the disease progression.
